# Integrating Multiple Correspondence Analysis and GWAS to Evaluate Reproductive Potential in Crossbred Heifers

**DOI:** 10.3390/genes17060611

**Published:** 2026-05-28

**Authors:** Andrés Rodríguez Serrano, Marcos Ahumada Velasco, Jesús María Cárdenas Beltrán, José Morelos Gómez

**Affiliations:** Profesional de Investigación, Universidad de Cartagena, Cartagena de Indias 130001, Colombia; camilo.rodriguez.serrano@gmail.com (A.R.S.); jcardenas@unisalle.edu.co (J.M.C.B.); jmorelosg@unicartagena.edu.co (J.M.G.)

**Keywords:** multiple correspondence analysis, GWAS, cattle genomics, animal reproduction

## Abstract

Objectives: The objective of this study was to evaluate the reproductive potential of crossbred heifers from dual-purpose systems using body conformation traits and to explore their genomic associations. Methods: A total of 522 heifers from the Colombian Caribbean region were phenotyped for structural and morphological traits, including body condition score, thoracic perimeter, height at withers, body length, ischium length, back level, hoof angle, stance width, hock angle, and rump level. Continuous variables were transformed into categorical classes and analyzed using multiple correspondence analysis (MCA) to build a reproductive potential index (RPI) that was used to perform a GWAS analysis to explore genomic regions. Results: The first two dimensions explained 11.6% and 8.2% of the total variation, respectively, and were used to construct an RPI. Heifers with higher RPI values exhibited greater thoracic perimeter, height, body length, and ischium length and were associated with wider chest and deeper body conformation, whereas lower RPI values were related to narrower and shallower body traits. However, some structurally desirable traits, such as centered stance, optimal hoof angle, and slightly sloped rump, were not clearly associated with high RPI. Genome-wide association analysis of the RPI did not reveal significant loci, although suggestive signals were identified on BTA3 and BTA19, near RSBN1, PHTF1, and WNT9B. Conclusions: These findings indicate that MCA-derived indices can summarize conformation-related variation in crossbred heifers, while the absence of strong associations suggests a polygenic architecture with small individual genetic effects.

## 1. Introduction

In cattle production systems, milk yield and average daily gain are often the primary selection targets. However, breeding programs must look beyond milk and meat output. Intensive and exclusive selection for milk yield in dairy cattle or for growth rate in beef cattle can negatively affect other economically important traits, particularly fertility [1,2]. For this reason, cattle are also evaluated using body conformation traits, which describe how closely an animal matches the structural ideal for its breed.

Conformation scoring contributes to the maintenance of good health and supports sustainable productivity. For example, correct udder structure improves milking efficiency; greater ischial separation and adequate rump width are associated with easier calving; and greater body capacity tends to improve metabolic stability, supporting earlier puberty, better ovarian activity, and improved conception rates. Additionally, well-formed legs and hooves are essential for supporting body weight, enabling long walking distances to access forage, and reducing the incidence of lameness [3]. These attributes also support the maintenance of an adequate body condition score (BCS), a key determinant of reproductive performance.

However, conformation scoring systems and their relationships with productivity have been evaluated primarily in taurine breeds, particularly Holstein cattle, which may limit their applicability in regions where most cattle populations are crossbred and present a high proportion of *Bos indicus* genetics, such as the Colombian Caribbean [4]. Nevertheless, linear evaluation systems developed for Zebu breeds, including Brahman and Gyr, provide an appropriate baseline for phenotypic assessment [5,6]. These systems include measurements of stature, body depth, thoracic perimeter, chest width, back and rump alignment, ischial width, hoof and hock angles, and overall leg conformation.

Selection of individuals based on body conformation traits can accelerate genetic progress in other economically important traits [7]. Body conformation is often one of the first criteria used in selection programs, particularly in heifers, because full productive and reproductive records are not yet available. Therefore, structural assessment provides an early indicator of functional capacity and long-term productive potential.

The development of high-density bovine genotyping platforms and the application of genome-wide association studies (GWAS) have enabled the identification of genomic regions associated with phenotypes of interest [8], and these approaches have increasingly been incorporated into animal breeding research. However, most GWAS have been conducted in purebred cattle populations, with comparatively fewer studies focusing on multibreed or crossbred populations, which represent the predominant cattle population in the Colombian Caribbean. GWAS can be combined with multiple correspondence analysis (MCA) by first constructing composite phenotypes that integrate categorical and continuous variables into a reduced set of latent dimensions used to score individuals. These MCA-derived scores can then be used as quantitative phenotypes in GWAS, where associations between individual SNPs and phenotypic variation are tested across the genome. Allelic variation at each SNP is evaluated to identify genomic regions statistically associated with the composite phenotype, which may harbor genes involved in the biological processes underlying the trait.

Therefore, the objective of this study was to construct a reproductive potential index based on body conformation traits in crossbred heifers from the Colombian Caribbean region using multiple correspondence analysis (MCA), and to explore genomic regions associated with this index through a genome-wide association study (GWAS). Additionally, this study contributes to improving the understanding of crossbred cattle populations, which remain underrepresented in genomic research compared to purebred populations.

## 2. Materials and Methods

The study was conducted in the Colombian Caribbean region, specifically in the departments of Bolívar, Córdoba, and Sucre. A total of 522 crossbred heifers were selected for the evaluation of body conformation traits, and tail hair collection for genomic analysis. Sampling was carried out during the mandatory cattle vaccination campaigns organized by the Colombian government (Foot and mouth disease and Brucellosis), between 10 and 20 May, during which producers temporarily confined their herds on their own farms. Animal confinement occurred only once per herd and lasted no longer than 20 min.

### 2.1. Heifers’ Selection

All heifers were selected from small-scale producers and presented a body weight between 300 and 350 kg (age between 18–24 months), at the time of assessment. All individuals were crossbred, with a predominance of *Bos indicus* genetic background, reflecting regional selection practices favoring animals adapted to local environmental conditions.

Heifers were selected from municipalities of Ciénaga de Oro, El Varal, Magangué, Montelíbano, Montería, San Carlos, and Tierralta which have an average temperature of 27 °C, annual precipitation of approximately 1500 mm, relative humidity exceeding 75%, and altitudes ranging from 0 to 100 m above sea level.

### 2.2. Body Conformation

Conformation traits were recorded as both categorical and continuous variables (Table 1). Categorical variables included back level, hoof angle class, stance width class, hock angle, and rump level, whereas continuous variables included body condition score (BCS), thoracic perimeter, height at withers, body length, and ischium length.

### 2.3. MCA

Continuous variables were transformed into categorical variables by classifying measurements into predefined structural categories. This transformation was performed because many conformation traits in cattle are traditionally recorded as categorical classes in structural evaluation systems. Multiple Correspondence Analysis (MCA) was then applied to explore the multivariate structure of the categorical body conformation traits using RStudio (v4.4.1) and the FactoMineR R package.

MCA was applied to identify latent dimensions summarizing the association patterns among categorical traits. Individual coordinates for the first two principal dimensions were extracted and used to describe structural variation among animals. To construct a composite phenotype for genomic analysis, the coordinates of the first two dimensions were standardized (z-scores). Based on the biological interpretation of these dimensions, a Reproductive Potential Index (RPI) was calculated by combining the standardized scores, generating a continuous variable that reflects overall structural adequacy. A composite Reproductive Potential Index (RPI) was then calculated by combining the standardized scores of the first two MCA dimensions:

$${RPI}_{i}=Z_{Dim1,i}+Z_{Dim2,i}$$

where RPI__i_ = reproductive potential index of the ith heifer; Z_Dim1,i_= standardized coordinate of dimension 1 and Z_Dim2,i_ = standardized coordinate of dimension 2. This index was subsequently used as the quantitative phenotype in the GWAS.

RPI values were categorized into three classes (low, medium, and high reproductive potential) based on terciles of the distribution. This approach allowed for a balanced classification of animals and facilitated the interpretation of structural differences among groups, while preserving the continuous nature of the index for subsequent analyses.

### 2.4. Genomic Analysis

DNA for genomic analysis was extracted from hair follicles obtained from tail hair samples. Hair collection was performed using a rapid upward pull to remove at least 20 hairs with intact follicles, which were stored in paper envelopes until processing. Genomic DNA was extracted using the MagMAX™ CORE Nucleic Acid Purification Kit (Thermo Fisher Scientific, Waltham, MA, USA). Genotyping and identification of commercially relevant genetic variants were performed using the Axiom™ Analysis Suite v4.0 (Thermo Fisher Scientific, Waltham, MA, USA) with the Axiom™ Bovine Genotyping 100K Array. Quality control filtering was conducted using PLINK v1.9 [9]. Markers were filtered based on the following criteria: call rate ≥ 95%, minor allele frequency (MAF) ≥ 0.05, Hardy–Weinberg equilibrium (HWE) *p*-value > 1 × 10^−6^, and missing genotype rate < 10%. Analyses were restricted to autosomal markers (*Bos taurus* chromosomes 1–29).

Genome-wide association (GWAS) was performed using a linear regression model implemented with the --linear option in PLINK V1.9, where each single nucleotide polymorphism (SNP) was tested individually for association with the quantitative trait. The Reproductive Potential Index (RPI), derived from the Multiple Correspondence Analysis (MCA), was used as the quantitative phenotype.

Manhattan plots for identification of significant SNPs were generated using the RSdtudio V4.4.1 package “qqman” with horizontal lines representing suggestive (*p* < 1 × 10^−5^) and genome-wide significance thresholds (*p* < 5 × 10^−8^), displayed as −log_10_(*p*) values. Gene annotation was performed using the Ensembl genome database for *Bos taurus* (ARS-UCD2.0 assembly). Gene coordinates and functional annotations were retrieved through the Ensembl REST API (https://www.ensembl.org/Bos_taurus/Info/Index) accessed on 12 February 2026.

### 2.5. Statistical Analysis

Continuous variables derived from body conformation measurements were analyzed using one-way analysis of variance (ANOVA). When significant effects were detected, Tukey’s honestly significant difference (HSD) test was used for multiple comparisons of means (*p* < 0.05).

## 3. Results

**Figure 1:**
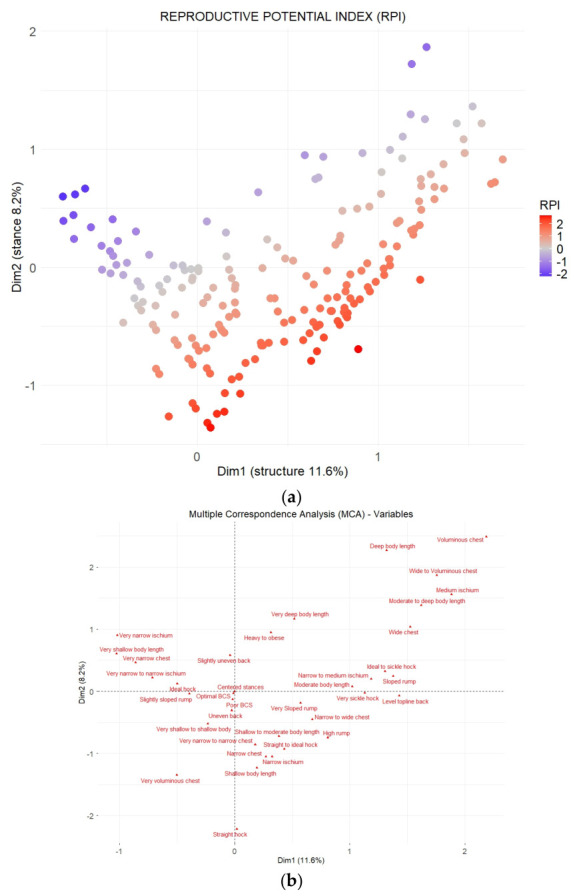
Reproductive potential index (RPI) for individuals (**a**) and MCA conformation traits (**b**).

Through Multiple Correspondence Analysis (MCA), heifers were classified into three categories of reproductive potential (high, medium, and low) according to the Reproductive Potential Index (RPI), which ranged from −2 to 2, with lower values indicating reduced potential (Figure 1a). The first and second dimensions explained 11.6% and 8.2% of the total variation, respectively. Heifers with higher RPI values were associated with conformation traits indicative of greater structural development, including voluminous chest, wide chest, deep body length, medium ischium length, straight hock, and well-aligned back. In contrast, heifers with lower RPI values were associated with traits reflecting reduced body capacity, such as narrow chest, narrow ischium length, and shallow body depth. However, not all traits considered optimal were strongly associated with higher RPI values. Centered stance, ideal hoof angle, optimal body condition score (BCS), and slightly sloped rump did not show a clear association with either high or low reproductive potential (Figure 1b).

According to better conformation traits, heifers with high and medium potential showed higher thoracic perimeter, height, and body and ischium length (Table 2). Differences between high- and medium-potential cows were not statistically significant, except for body condition score (BCS) and ischial length. Overall, these groups were 16.2 cm wider, 3.5 cm taller, 16.8 cm deeper, and 4.0 cm broader compared with low-potential cows.

**Figure 2:**
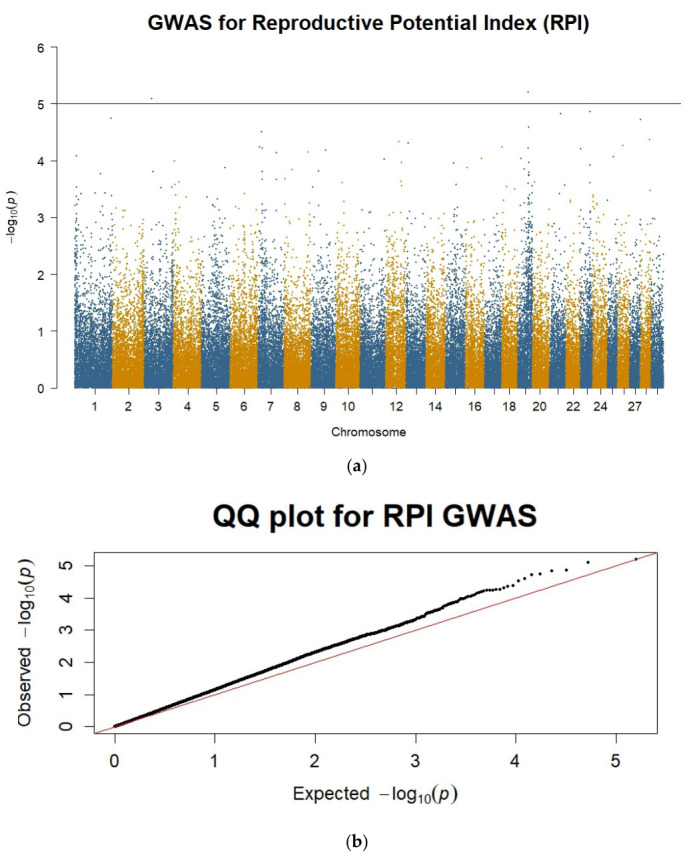
Manhattan (**a**) and quantile–quantile plot (**b**) from the GWAS of the reproductive potential index in crossbred heifers.

Genome-wide association analysis of the RPI did not reveal significant genotypic associations across the autosomes. Only BTA 3 and 19 showed SNPs surpassing the suggestive threshold (*p* < 1 × 10^−5^) (Figure 2a). These signals were limited and did not form extended genomic regions, suggesting the absence of major effect loci underlying the trait. This interpretation is consistent with the QQ plot, in which most observed *p*-values closely followed the expected null distribution. A moderate deviation was observed only in the extreme tail of the distribution (Figure 2b), corresponding to the small number of SNPs identified in the Manhattan plot.

Two significant SNPs were detected to RPI, one in BTA 3 in the position (bp) 29,391,380 rs136606137, with the associated genes RSBN1 and PHTF1, and one in the BTA 19 in the position (bp) 45,081,038 rs136606137 with the associated gene WNT9B.

## 4. Discussion

However, although good conformation traits were associated with the Reproductive Potential Index (RPI) derived from the MCA, not all high-potential cows presented optimal conformation, particularly in traits such as stance quality and hoof and hock angles. This may be related to the fact that the study was conducted using crossbred animals, from dual-purpose systems in which selection for specific conformation traits is not the primary objective, as producers tend to prioritize body size, production and their capacity to conceive and maintain calves [10]. In multiracial individuals, it is difficult to fit them into an ideal body type, since uncontrolled crossing may result in the incomplete expression of adaptive and productive advantages expected from mating [11,12].

Multivariate analyses have been used to describe body conformation in cattle. The authors of [13] demonstrated the potential of these approaches for breeding and selection programs by reducing the number of body measurements required to characterize White Fulani cattle. Several of the measurements evaluated in that study are comparable to those used in the present work. The authors also emphasized the importance of evaluating animals within a similar age range, since body traits differ significantly between younger and older animals, which may affect the interpretation of conformation patterns.

Most studies evaluating conformation traits and their relationship with productive and reproductive performance have been conducted in *Bos taurus* breeds, whereas fewer studies have been carried out in *Bos indicus* populations, mainly in Gyr cattle [3,14]. In crossbred animals from dual-purpose systems, research on conformation traits is more limited, as structural evaluations are typically performed within the context of purebred breeding programs [15].

In the present study, heifers classified with high reproductive potential showed greater thoracic perimeter, height, body length, and ischium length measurements. However, several studies have reported weak correlations between conformation traits and production or reproductive performance. Ref. [16] reported correlations of 0.16 and −0.0024 between chest width and the udder central ligament, a structure considered important for supporting high milk production. Similarly, Ref. [17] found low correlations (−0.01 to 0.03) between stature, chest width, angularity, rump angle, and foot angle with reproductive indicators such as calving interval and services per conception. Despite these low correlations, adequate body conformation remains important for animal functionality and welfare. For example, proper leg conformation has been shown to be a strong predictor of functional longevity in dairy cattle [18]. In addition, Ref. [19] reported positive correlations between production traits and productive life in Holstein cattle, where the general score for type and conformation, including body depth, chest width, rump angle, udder attachment, and leg alignment, showed correlations of 0.22 with productive performance and 0.08 with productive life. These findings highlight the importance of incorporating optimal conformation traits into selection programs.

The absence of strong genome-wide associations suggests that the Reproductive Potential Index (RPI), derived from conformation traits, may be influenced by multiple genes with relatively small individual effects; additionally, the absence of significant associations may be due to insufficient sample size, as genetically heterogeneous crossbred populations typically require larger sample sizes to achieve adequate statistical power for detecting genomic effects. These results differ from those reported by the authors of [20], who identified 63 significant SNP loci and 66 candidate genes associated with traits such as body size, feet and legs, and udder conformation in Chinese Holstein cattle. Similar findings were reported in [21] in North American Holstein populations, where significant SNPs associated with conformation traits were detected across most chromosomes. In addition, Ref. [3] identified three annotated genes on BTA6 associated with body height (FAM13A, NAPIL5 and HERC3) and two annotated genes on BTA1 associated with rump angle (CMSS1 and TMEM30) in Gyr dairy cattle; these are different to those reported in the present study, where annotated genes were RSBN1 and PHTF1 in BTA 3, which have been associated with immunological processes, disease resistance and fertility [22,23], and WNT9B in the BTA 19, which have been associated with adult mammary gland fat [24].

## 5. Conclusions

Individuals with higher scores in the index showed better conformation measurements, particularly in body size and ischium length. However, well-aligned stances and hoof conformation were not strictly associated with the highest-ranked heifers. This may be explained by the multiracial composition of the evaluated population, as crossbred animals may not conform to the ideal body type typically defined for specific breeds. Nevertheless, the selection of replacement heifers for breeding programs should consider structural soundness, particularly well conformed stances, which are essential in extensive production systems where animals must walk long distances in search of forage and water. These traits, which were not consistently associated with high RPI values, limit the utility of the implemented methodology. However, further analyses in more structurally uniform populations, such as purebred animals, could provide a more comprehensive validation of the proposed approach.

The reproductive potential index and its component traits did not show strong genome wide associations, and only three annotated genes were identified. This suggests that the evaluated conformation traits may be controlled by multiple genes with relatively small individual effects, which are consistent with the polygenic nature of most complex traits. In addition, GWAS analyses tend to detect stronger associations in populations subjected to high levels of genetic selection. In contrast, crossbred animals from dual-purpose systems in the Colombian Caribbean represent a heterogeneous genetic background and constitute an important component of the national cattle inventory. Therefore, future studies could include bigger sample size and single-trait GWAS analyses to identify candidate genes associated with specific conformation traits, which may contribute to the development of more effective genetic improvement programs.

## Figures and Tables

**Table 1:** Variables evaluated for body conformation traits.

Variable	Description	Scale	Class
BCS	Visual measurement of fat reserves and nutritional status	1–3	Poor condition
		4–6	Moderate—optimal
		7–9	Heavy—obese
Thorax perimeter (cm)	Circumference of the chest immediately behind the forelimbs	<184	Very narrow
		184–197	Narrow to wide
		198–206	Wide to voluminous
		>206	Very voluminous
Height (cm)	Vertical distance from the ground to the highest point of the withers	<126	Short
		126–135	Medium
		>136	Tall
Length (cm)	Distance measured from the point of the shoulder (cranial humeral tuberosity) to the point of the ischium (pin bone)	<155	Very shallow
		155–166	Shallow to medium
		167–178	Medium to long
		>178	Very long
Back level	Alignment of the heifer’s topline from the withers to the rump		Uneven
			Slightly uneven
			Level topline
Hoof angle	Inclination of the hoof wall relative to the ground	<15–30°	Low angle
		31–53°	Ideal
		>54°	High angle
Stance width	Lateral spacing between the limbs when the animal is standing		Very close
			Close
			Centered
			Wide
Hock angle	Degree of angulation at the hock joint when the animal is viewed from the side		Straight
			Ideal
			Sickle
			Very Sickle
Rump level	Inclination of the pelvic region from the hips to the pin bones		High
			Very sloped
			Sloped
			Slightly sloped
Ischium length (cm)	Distance between the hip region and the pin bones (ischial tuberosities)	<25	Very narrow
		25–32	Narrow
		33–37	Medium
		>37	Wide

**Table 2:** Conformation measurements for crossbred heifers.

Group	BCS	Thorax Perimeter	Height	Length	Ischium Length
High potential	5.3 ± 0.5 ^c^*	186.5 ± 3.7 ^a^	134.9 ± 4.4 ^a^	158.6 ± 3.9 ^a^	26.5 ± 1.5 ^a^
Medium potential	5.7 ± 0.6 ^b^	185.7 ± 8.2 ^a^	134.8 ± 4.0 ^a^	158.5 ± 7.8 ^a^	25.5 ± 2.8 ^b^
Low potential	6.0 ± 0.1 ^a^	169.9 ± 7.8 ^b^	131.3 ± 4.9 ^b^	141.7 ± 6.6 ^b^	22.0 ± 1.7 ^c^

* Means with different letters within a column differ significantly according to Tukey’s HSD test (*p* < 0.05).

## Data Availability

The dataset is not publicly available because it is treated as confidential information.
